# Quantitative parameters of lymphocyte nuclear morphology in bronchoalveolar lavage fluid as novel biomarkers for sarcoidosis

**DOI:** 10.1186/s13023-021-01926-x

**Published:** 2021-07-03

**Authors:** Yasushi Horimasu, Kakuhiro Yamaguchi, Shinjiro Sakamoto, Takeshi Masuda, Shintaro Miyamoto, Taku Nakashima, Hiroshi Iwamoto, Kazunori Fujitaka, Hironobu Hamada, Noboru Hattori

**Affiliations:** Department of Respiratory Medicine, Hiroshima University Hospital, 1-2-3 Kasumi, Minami-ku, Hiroshima City, Hiroshima 734-8551 Japan

**Keywords:** Interstitial lung disease, Lymphocytosis, Nuclear area, Nuclear perimeter, Radius ratio, Roundness

## Abstract

**Background:**

Bronchoalveolar lavage (BAL) is one of the fundamental examinations for the differential diagnosis of interstitial lung diseases (ILDs), and lymphocytosis strongly indicates alternative diagnoses rather than idiopathic pulmonary fibrosis. However, the BALF lymphocytosis is observed in several ILDs. We considered that quantitative evaluation of the BALF lymphocyte nuclear morphology would be useful in the differential diagnosis of ILDs with increased BALF lymphocyte fraction.

**Results:**

One hundred and twenty-one patients with ILDs having increased BALF lymphocyte fraction were recruited (68 in the development cohort and 53 in the validation cohort). In the development cohort, BALF lymphocyte nuclei in sarcoidosis patients showed significantly smaller areas, shorter perimeters, lower radius ratios, and increased roundness than those of other ILD patients (p < 0.001 for each). Next, the fractions of lymphocytes with small areas, short perimeters, low radius ratios, and increased roundness, which were determined based on receiver operating characteristic (ROC) analyses-based thresholds, were demonstrated to be higher in sarcoidosis patients than in the other ILD patients (p < 0.001 for each). Furthermore, when we combined size-representing parameters with shape-representing parameters, the fraction of lymphocytes with small and round nuclei showed approximately 0.90 of area under the ROC curve in discriminating sarcoidosis both in the development cohort and the validation cohort.

**Conclusion:**

This study is the first to demonstrate the usefulness of quantitative parameters of BALF lymphocyte nuclear morphology as novel biomarkers for sarcoidosis.

**Supplementary Information:**

The online version contains supplementary material available at 10.1186/s13023-021-01926-x.

## Background

Bronchoalveolar lavage (BAL) is a fundamental examination performed as part of a bronchoscopy procedure, mainly for the differential diagnosis of diffuse lung diseases. The total cell count and differential cell count of BAL fluid (BALF) as well as the analysis of BALF supernatant content, such as several cytokines, protein biomarkers, and microorganism antigens, have been recognized as supportive information for the diagnosis of several lung diseases.

However, the clinical utility of BAL in the diagnosis of interstitial lung disease (ILD) remains controversial. In 2012, the American Thoracic Society published the official guideline, in which BAL was recommended only when high-resolution computed tomography (HRCT) findings were not diagnostic [1]. However, Ohshimo et al. reported that even in those with HRCT findings typical for idiopathic pulmonary fibrosis (IPF), 8% of patients showed BALF lymphocytosis, and all of them finally reached alternative diagnoses [2]. In this context, some arguments about the utility of BAL in the diagnosis of ILDs have arisen [3, 4].

Most recently, the results of two systematic reviews have been reported. Adderley reported that the proportion of BAL lymphocytes was 42.8% in patients with chronic hypersensitivity pneumonia (CHP) and 10.0% in IPF patients [5]. In addition, Patolia et al. reported that a threshold of 20% BALF lymphocyte fraction could distinguish fibrotic HP from IPF, with sensitivity and specificity higher than 60% [6].

Based on these findings, BAL is still important in the diagnoses of HP, sarcoidosis, and connective tissue disease-associated ILD (CTD-ILD), especially when we cannot be fully confident in the diagnosis of IPF. However, the finding of the elevated lymphocyte fraction in BALF is not enough for a detailed differential diagnosis, as there are several ILDs that cause increased BALF lymphocyte fraction. More importantly, such ILDs with BALF lymphocytosis substantially vary in their clinical manifestations and prognostic characteristics. Therefore, differential diagnosis among these ILDs is of critical importance. To support the differential diagnosis among ILDs with increased BALF lymphocyte fraction, the CD4 to CD8 ratio is clinically available. In addition, there is a report demonstrating the BALF lymphocyte nuclear morphological differences between sarcoidosis and HP [7], although no quantitative approach has been reported.

## Results

### Morphological parameters of BALF lymphocyte nuclei in sarcoidosis patients significantly differed from those in the other ILD patients

**Fig. 1:**
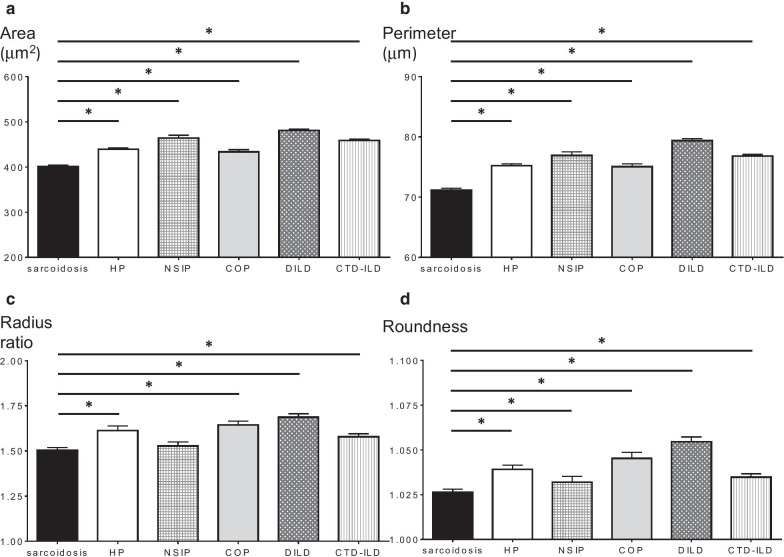
Area, perimeter, radius ratio, and roundness of BALF lymphocyte nuclei. **a** Areas of BALF lymphocyte nuclei in patients with sarcoidosis, HP, NSIP, COP, DILD, and CTD-ILD were 400.97 ± 2.92 μm^2^, 439.11 ± 3.10 μm^2^, 464.19 ± 6.24 μm^2^, 433.61 ± 4.81 μm^2^, 480.81 ± 3.24 μm^2^, and 458.61 ± 2.97 μm^2^, respectively. **b** Perimeters of BALF lymphocyte nuclei in patients with sarcoidosis, HP, NSIP, COP, DILD, and CTD-ILD were 71.18 ± 0.30 μm, 75.21 ± 0.31 μm, 76.92 ± 0.58 μm, 75.07 ± 0.47 μm, 79.39 ± 0.32 μm, and 76.81 ± 0.28 μm, respectively. **c** Radius ratios of BALF lymphocyte nuclei in patients with sarcoidosis, HP, NSIP, COP, DILD, and CTD-ILD were 1.50 ± 0.014, 1.61 ± 0.026, 1.53 ± 0.023, 1.64 ± 0.021, 1.69 ± 0.019, and 1.58 ± 0.016, respectively. **d** Roundness of BALF lymphocyte nuclei in patients with sarcoidosis, HP, NSIP, COP, DILD, and CTD-ILD was 1.026 ± 0.0018, 1.039 ± 0.0023, 1.032 ± 0.0034, 1.045 ± 0.0035, 1.055 ± 0.0027, and 1.035 ± 0.0020, respectively. The error bars represent the standard error of the mean. *p value is under the significance level set at α = 0.010 (five comparisons in six groups). BALF, bronchoalveolar lavage fluid; HP, hypersensitivity pneumonia; NSIP, nonspecific interstitial pneumonia; COP, cryptogenic organizing pneumonia; DILD, drug-induced interstitial lung disease; CTD-ILD, connective-tissue disease associated interstitial lung disease

As shown in Fig. 1, in the development cohort, BALF lymphocyte nuclear areas in sarcoidosis patients (400.97 ± 2.92 μm^2^) were significantly smaller than those in the other ILD patients. The BALF lymphocyte nuclear perimeter in sarcoidosis patients (71.18 ± 0.30 μm) was significantly shorter than that in the other ILD patients. The BALF lymphocyte nuclear radius ratio in sarcoidosis patients (1.50 ± 0.014) was significantly lower than that in the other ILD patients. Furthermore, BALF lymphocyte nuclear roundness in sarcoidosis patients (1.026 ± 0.0018) was significantly higher than that in the other ILD patients. Table 1 shows the correlations among the four morphological parameters. All four parameters significantly correlated with each other, and more importantly, the correlation coefficients were extremely high between area and perimeter as well as between radius ratio and roundness, whereas they were relatively low for the other correlations (Table 1). These results may demonstrate a strong correlation between area and perimeter, which can be considered as size-representing parameters, as well as between the radius ratio and roundness, which can be considered as shape-representing parameters.

**Table 1:**
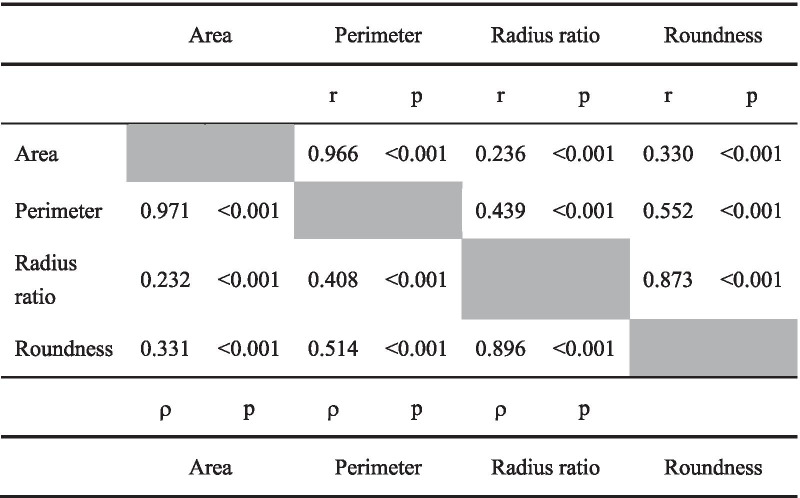
Correlations between the four morphological parameters

The cells to the upper right of the diagonal line show Pearson correlation coefficients, and the cells to the lower left of the diagonal line show Spearman’s rank correlation coefficients

### The sarcoidosis-diagnostic threshold for each morphological parameter of BALF lymphocyte nucleus was determined in accordance with the ROC curves

**Fig. 2:**
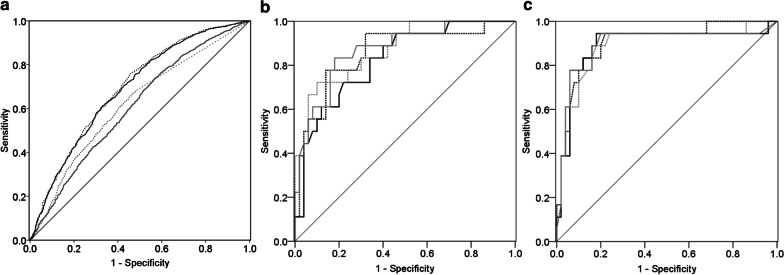
ROC curves for the four morphological parameters. **a** ROC curves were drawn for nuclear area (solid black line, AUROC = 0.689, 95% confidence interval (CI) 0.668–0.710, p < 0.001), perimeter (dotted black line, AUROC = 0.695, 95% CI 0.674–0.716, p < 0.001), radius ratio (solid gray line, AUROC = 0.607, 95% CI 0.585–0.629, p < 0.001), and roundness (dotted gray line, AUROC = 0.619, 95% CI 0.598–0.640, p < 0.001). **b** ROC curves were drawn for the fraction of lymphocytes with small nuclei (solid black line, AUROC = 0.824, 95% CI 0.717–0.932, p < 0.001), short nuclear perimeters (dotted black line, AUROC = 0.856, 95% CI 0.749–0.964, p < 0.001), low nuclear radius ratios (solid gray line, AUROC = 0.870, 95% CI 0.775–0.965, p < 0.001), and increased nuclear roundness (dotted gray line, AUROC = 0.871, 95% CI 0.778–0.964, p < 0.001). (c) ROC curves were drawn for the fraction of lymphocytes with small nuclear areas and low radius ratios (solid black line, AUROC = 0.892, 95% CI 0.783–1.000, p < 0.001), with small nuclear areas and increased roundness (dotted black line, AUROC = 0.904, 95% CI 0.819–0.989, p < 0.001), with short nuclear perimeters and low radius ratios (solid gray line, AUROC = 0.894, 95% CI 0.784–1.000, p < 0.001), and with short nuclear perimeters and increased roundness (dotted gray line, AUROC = 0.890, 95% CI 0.790–0.990, p < 0.001). ROC, receiver operating characteristic; AUROC: area under the ROC curve

In order to evaluate the discriminating ability of area, perimeter, radius ratio, and roundness of BALF lymphocyte nuclei in distinguishing between sarcoidosis and the other ILDs, receiver operating characteristic (ROC) curves were drawn (Fig. 2a). It was revealed that their discriminating ability was statistically significant; however, area under the ROC curve (AUROC) was not sufficiently high, namely 0.689, 0.695, 0.607, and 0.619 for area, perimeter, radius ratio, and roundness, respectively (Fig. 2a). Based on these ROC curves, the sarcoidosis-diagnostic thresholds of nuclear area, nuclear perimeter, radius ratio, and roundness were determined as 428.1 μm^2^, 71.3 μm, 1.346, and 1.0057, respectively. Lymphocytes with nucleus presenting values below these thresholds were deemed to have small areas, short perimeters, low radius ratios, or increased roundness. Additionally, frequency distribution charts for the four morphological parameters of BALF lymphocyte nuclei accompanied by representative lymphocytes are shown in Additional file 1: Figs. S1, S2, S3, and S4.

### The combination of size-representing parameters and shape-representing parameters of BALF lymphocyte nuclei is useful to discriminate sarcoidosis from the other ILDs

**Fig. 3:**
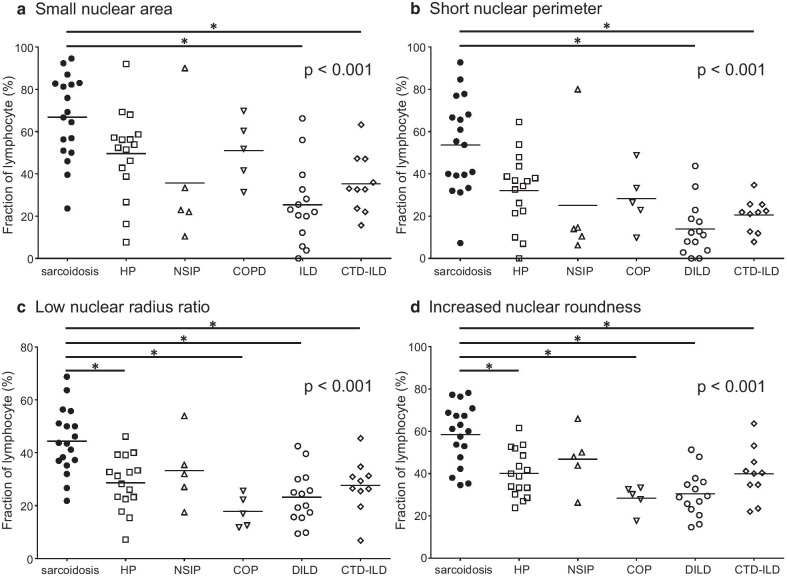
Fractions of lymphocyte with small or round nucleus. **a** The fractions (%) of lymphocytes with small nuclear areas in sarcoidosis, HP, NSIP, COP, DILD, and CTD-ILD were 66.8 ± 4.7, 49.6 ± 5.1, 35.7 ± 1.4, 51.0 ± 6.8, 25.4 ± 5.0, and 35.3 ± 4.4, respectively. **b** The fractions (%) of lymphocytes with short nuclear perimeters in sarcoidosis, HP, NSIP, COP, DILD, and CTD-ILD were 53.7 ± 5.3, 32.1 ± 4.3, 25.1 ± 1.4, 28.3 ± 6.4, 14.0 ± 3.4, and 20.6 ± 2.5, respectively. **c** The fractions (%) of lymphocytes with low nuclear radius ratios in sarcoidosis, HP, NSIP, COP, DILD, and CTD-ILD were 44.4 ± 2.9, 28.6 ± 2.6, 33.2 ± 6.0, 17.8 ± 2.7, 23.2 ± 2.7, and 27.6 ± 3.2, respectively. **d** The fractions (%) of lymphocytes with increased nuclear roundness in sarcoidosis, HP, NSIP, COP, DILD, and CTD-ILD were 58.4 ± 3.4, 40.1 ± 2.8, 46.8 ± 6.4, 28.3 ± 2.8, 30.4 ± 2.9, and 39.9 ± 4.0, respectively. The horizontal bars represent the mean values. The p value for the Kruskal–Wallis test is shown on the upper right corner of each chart. *the p value for the Mann–Whitney U test is under a significance level set at α = 0.010 (five comparisons in five groups). HP, hypersensitivity pneumonia; NSIP, nonspecific interstitial pneumonia; COP, cryptogenic organizing pneumonia; DILD, drug-induced interstitial lung disease; CTD-ILD, connective-tissue disease associated interstitial lung disease

**Fig. 4:**
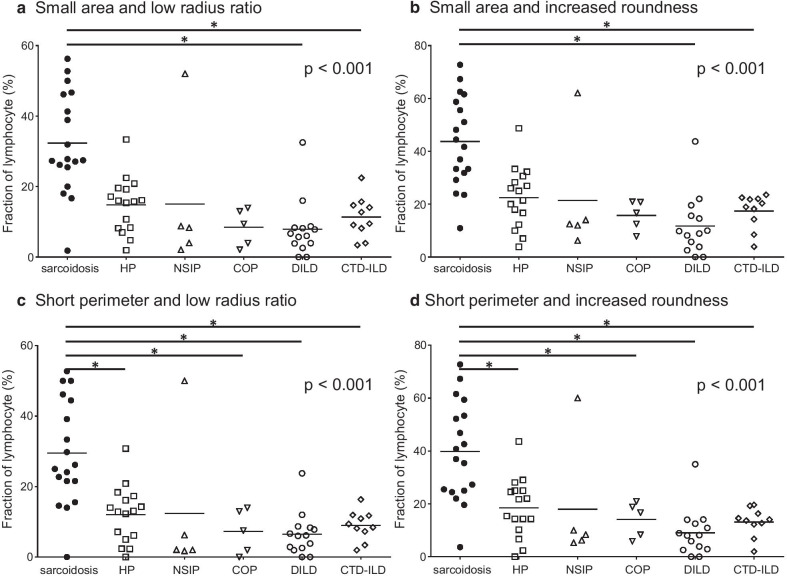
Fractions of lymphocytes with small and round nuclei. **a** The fractions (%) of lymphocytes with small nuclear areas and low radius ratios in sarcoidosis, HP, NSIP, COP, DILD, and CTD-ILD were 32.3 ± 3.4, 14.8 ± 2.0, 15.0 ± 9.3, 8.5 ± 2.4, 7.9 ± 2.2, and 11.4 ± 1.8, respectively. **b** The fractions (%) of lymphocytes with small nuclear areas and increased roundness in sarcoidosis, HP, NSIP, COP, DILD, and CTD-ILD were 43.7 ± 4.0, 22.5 ± 2.9, 21.4 ± 1.0, 15.7 ± 2.5, 11.8 ± 3.1, and 17.4 ± 2.1, respectively. **c** The fractions (%) of lymphocytes with short nuclear perimeters and low radius ratios in sarcoidosis, HP, NSIP, COP, DILD, and CTD-ILD were 29.5 ± 3.5, 12.1 ± 2.0, 12.4 ± 9.4, 7.3 ± 2.8, 6.5 ± 1.6, and 9.0 ± 1.3, respectively. **d** The fractions (%) of lymphocyte with short nuclear perimeters and increased roundness in sarcoidosis, HP, NSIP, COP, DILD, and CTD-ILD were 39.8 ± 4.4, 18.5 ± 2.8, 18.0 ± 11.0, 14.1 ± 3.0, 90.4 ± 2.4, and 13.1 ± 1.7, respectively. The horizontal bars represent the mean values. The p value for the Kruskal–Wallis test is shown on the upper right corner of each chart. *The p value for the Mann–Whitney U test is under a significance level set at α = 0.010 (five comparisons in five groups). HP, hypersensitivity pneumonia; NSIP, nonspecific interstitial pneumonia; COP, cryptogenic organizing pneumonia; DILD, drug-induced interstitial lung disease; CTD-ILD, connective-tissue disease associated interstitial lung disease

As shown in Fig. 3, the fraction of lymphocytes with small nuclear areas, short nuclear perimeters, low radius ratios, and increased roundness tended to be higher in sarcoidosis patients than in the other ILD patients. In addition, sufficient discriminating abilities of the fraction of lymphocytes with small nuclear areas, short nuclear perimeters, low radius ratios, and increased roundness were demonstrated by the four ROC curves with an AUROC of over 0.80 (Fig. 2b). Furthermore, we aimed to establish a more useful parameter for distinguishing sarcoidosis by combining size-representing parameters with shape-representing parameters as follows: nuclear area and radius ratio, nuclear area and roundness, nuclear perimeter and radius ratio, and nuclear perimeter and roundness. As shown in Fig. 4, regardless of the combination of parameters, the fraction of lymphocytes with small and round nuclei was significantly higher in sarcoidosis patients than in the other ILD patients. Importantly, excellent discriminating abilities of the fractions of lymphocytes with small nuclear areas and low radius ratios, small nuclear areas and increased roundness, short nuclear perimeters and low radius ratios, and short nuclear perimeters and increased roundness were demonstrated by the four ROC curves with an AUROC of approximately 0.90 (Fig. 2c).

### The fraction of lymphocytes with small and round nuclei in sarcoidosis patients showed no significant correlations with the BALF CD4/CD8 ratio

**Fig. 5:**
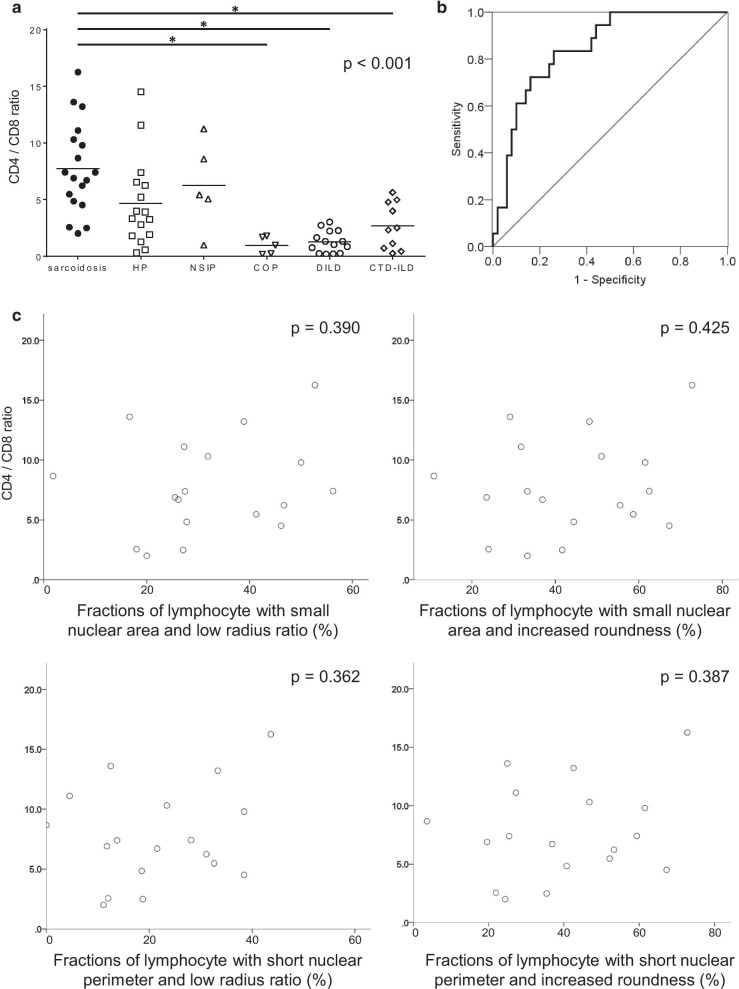
BALF CD4/CD8 ratios in sarcoidosis and in the other ILDs. **a** BALF CD4/CD8 ratios in sarcoidosis, HP, NSIP, COP, DILD, and CTD-ILD were 7.75 ± 0.95, 4.66 ± 0.98, 6.25 ± 1.7, 0.960 ± 0.34, 1.28 ± 0.26, and 2.67 ± 0.65, respectively. The horizontal bars represent the mean values. The p value for the Kruskal–Wallis test is shown on the upper right corner. * the p value for the Mann–Whitney U test is under a significance level set at α = 0.010 (five comparisons in five groups). **b** The ROC curve for the BALF CD4/CD8 ratio revealed an AUROC of 0.844 (95% confidence interval 0.749–0.940, p < 0.001) in distinguishing sarcoidosis. **c** Scatterplots representing the correlations between CD4/CD8 ratio and the fraction of lymphocytes with small and round nuclei defined as follows: small nuclear area and low radius ratio (upper left, p = 0.390, R^2^ = 0.047), small nuclear area and increased roundness (upper right, p = 0.425, R^2^ = 0.040), short nuclear perimeter and low radius ratio (lower left, p = 0.362, R^2^ = 0.052), and short nuclear perimeter and increased roundness (lower right, p = 0.387, R^2^ = 0.047). HP, hypersensitivity pneumonia; NSIP, nonspecific interstitial pneumonia; COP, cryptogenic organizing pneumonia; DILD, drug-induced interstitial lung disease; CTD-ILD, connective-tissue disease associated interstitial lung disease; ROC, receiver operating characteristic; AUROC: area under the ROC curve

In the development cohort, the BALF CD4/CD8 ratio was significantly higher in sarcoidosis patients than in the other ILD patients (Fig. 5a). The AUROC for the CD4 to CD8 ratio was 0.844, which was significantly high in distinguishing between sarcoidosis and the other ILDs (Fig. 5b). In addition, the AUROC for the CD4 to CD8 ratio showed a significant difference with none of the following: the AUROC of the fraction of lymphocytes with small nuclear area and low radius ratio (p = 0.517), small nuclear area and increased roundness (p = 0.345), short nuclear perimeter and low radius ratio (p = 0.494), and short nuclear perimeter and increased roundness (p = 0.510). To further evaluate the associations between the nuclear morphological parameters of BALF lymphocytes and the clinical features of sarcoidosis, we investigated the correlation between the BALF CD4/CD8 ratio and the fraction of lymphocytes with small and round nuclei in sarcoidosis patients. As shown in Fig. 5c, the fraction of lymphocytes with small and round nuclei showed no significant correlation with the BALF CD4/CD8 ratio.

### The discriminating ability of the fraction of lymphocytes with small and round nuclei was reconfirmed in the validation cohort

Based on the ROC curve shown in Fig. 2c, the sarcoidosis-diagnostic cutoff value for the fraction of lymphocytes with small nuclear area and low radius ratio, small nuclear area and increased roundness, short nuclear perimeter and low radius ratio, and short nuclear perimeter and increased roundness were 16.4%, 23.0%, 14.0%, and 21.9%, respectively. As shown in Table 2, the sensitivity and specificity for each of these cutoff values in the development cohort were sufficiently high, and more importantly, those in the validation cohort were mostly comparable to their counterparts in the development cohort. Furthermore, in the validation cohort, the AUROC for the fraction of lymphocytes with small nuclear areas and low radius ratios, with small nuclear areas and increased roundness, with short nuclear perimeters and low radius ratios, and with short nuclear perimeters and increased roundness was 0.938, 0.922, 0.933, and 0.928, respectively (Table 2).

**Table 2:** Discriminating ability of the fractions of lymphocytes with small and round nuclei

	Fractions of lymphocytes with			
	small area and low radius ratio	small area and increased roundness	short perimeter and low radius ratio	short perimeter and increased roundness
Cutoff value	16.4%	23.0%	14.0%	21.9%
*Development cohort*				
Sensitivity	0.944	0.944	0.944	0.889
Specificity	0.820	0.780	0.800	0.820
Accuracy	0.853	0.824	0.838	0.838
*Validation cohort*				
AUROC (95% CI.)	0.938 (0.865–1.000)	0.922 (0.847–0.998)	0.933 (0.857–1.000)	0.928 (0.832–1.000)
Sensitivity	1.000	1.000	1.000	1.000
Specificity	0.711	0.622	0.733	0.711
Accuracy	0.755	0.679	0.774	0.755

AUROC, area under the ROC curve; CI, confidence interval

## Discussion

In the present study, we have demonstrated that the morphological parameters of BALF lymphocyte nuclei, namely area, perimeter, radius ratio, and roundness, can be novel diagnostic biomarkers for distinguishing between sarcoidosis and other ILDs. In particular, when used in combination, these parameters showed sufficiently high discriminating ability with an AUROC of approximately 0.90. Furthermore, the usefulness of these morphological parameters was confirmed in an independent validation cohort. This study is the first to demonstrate the utility of a quantitative assessment of BALF lymphocyte nuclear morphology in the differential diagnosis of diffuse lung diseases. When we found BALF lymphocytosis, the detailed differential diagnosis is of critical importance, because ILDs with BALF lymphocytosis substantially vary in their clinical manifestations and prognostic characteristics. We believe that the morphological parameters of BALF lymphocyte nuclei should be useful in the detailed differential diagnosis among ILDs with BALF lymphocytosis.

The most important finding in the present study is that the fraction of lymphocytes with small and round nuclei defined by the combinations of size-representing parameters and shape-representing parameters showed significant discriminating ability in distinguishing between sarcoidosis and the other ILDs. Although morphological differences in BALF lymphocyte nuclei between sarcoidosis and HP have already been reported [7], the strength of the present study is that we quantitatively assessed such differences and successfully demonstrated that the morphological parameters of BALF lymphocyte nuclei can be used as diagnostic biomarkers in the differential diagnosis of diffuse lung diseases. It has been reported that the alteration of nuclear size and shape is associated with changes in the composition of the nuclear pore complex [8, 9], changes in the expression of nuclear lamin isoform [8, 10], and the differential expression of the endoplasmic reticulum structural proteins [8, 11]. Therefore, nuclear size and shape may reflect nucleocytoplasmic transport status, chromatin organization, DNA rearrangement, amplification, and metabolism [8].

To date, a number of studies focusing on gene expression and proteome analyses have revealed the varied expression properties of numerous genes and proteins in various types of ILDs, including sarcoidosis [12–18]. Such differences in the gene and/or protein expression properties of various cytokines, chemokines, and other transcriptional factors in immune reactions or inflammatory responses can cause differences in nucleocytoplasmic transport status, chromatin organization, and DNA amplification, resulting in the alteration of nuclear size and shape between sarcoidosis and the other ILDs.

In addition, we also demonstrated that the AUROC of the fractions of lymphocytes with small and round nuclei was comparable to that of the BALF CD4/CD8 ratio, which is widely used clinically to support the differential diagnosis between sarcoidosis and other ILDs. However, as there was no significant correlation between the fractions of lymphocytes with small and round nuclei and the BALF CD4/CD8 ratio in sarcoidosis patients (Fig. 5c), it can be inferred that the difference in BALF lymphocyte nuclear size and shape does not reflect a difference in the lymphocyte population.

It is noteworthy that the discriminating ability of the fraction of lymphocytes with small and round nuclei was reconfirmed in the validation cohort, which was prospectively established including patients with various types of diagnosis. Importantly, the AUROC of the fractions of lymphocytes with small and round nuclei was over 0.90 in the validation cohort, although the accuracy for the sarcoidosis-diagnostic cutoff values, which were determined in the development cohort, was relatively low in the validation cohort (Table 2). These findings may indicate that the appropriate cutoff values of the fractions of lymphocytes with small and round nuclei need to be determined based on further prospective investigations with larger sample sizes.

Despite the novel and promising findings of the present study, we acknowledge that there are some limitations. First, only patients with BALF lymphocytosis, namely those with 20% or more of the lymphocyte fraction in BALF, were included in this study. We excluded those without BALF lymphocytosis in order to avoid the enormous effort required to analyze many lymphocytes in patients with low lymphocyte fractions. Therefore, it is unclear whether the fraction of lymphocytes with small and round nuclei can be useful biomarkers for distinguishing between sarcoidosis and other ILDs, even among those without BALF lymphocytosis. Additionally, no healthy control was included in this study because we do not perform BAL for healthy subjects mainly for the ethical reason. Second, the analysis of lymphocyte nuclear morphology depended on manual tracing of the outline of lymphocyte nuclei. Thus, there may be some inaccuracies in the detailed perception of lymphocyte nuclear morphology. However, we believe that such inaccuracies do not diminish the importance of our results because the discriminating ability of the fraction of lymphocytes with small and round nuclei was reconfirmed in the independent validation cohort. Third, this procedure of quantification of BALF lymphocyte nuclear morphology requires a large amount of effort. Therefore, it would be beneficial to explore the use of AI techniques in this field.

## Conclusions

In conclusion, following the quantitative analysis of the morphological features of BALF lymphocyte nuclei, the present study has successfully demonstrated that the fractions of lymphocytes with small and round nuclei in BALF can be novel diagnostic biomarkers for distinguishing between sarcoidosis and other ILDs.

## Methods

### Aim of this study

We believe that quantitative evaluation of BALF lymphocyte nuclear morphology will be useful for the differential diagnosis of ILDs with increased BALF lymphocyte fraction. We conducted the present study to evaluate the utility of the fraction of lymphocytes with small and round nuclei, which was defined in a quantitative manner, in the differential diagnosis of ILDs with increased BALF lymphocyte fraction.

### Characteristics of the subjects

We have established the development cohort in a retrospective manner and comprised sixty-eight patients with a lymphocyte fraction of 20% or more in BALF diagnosed as either of the following: sarcoidosis, HP, nonspecific interstitial pneumonia, cryptogenic organizing pneumonia, drug-induced ILD, or CTD-ILD. The clinical characteristics of the development cohort are presented in Table 3. The validation cohort was established in a prospective manner and comprised 53 patients, regardless of diagnosis, who showed a lymphocyte fraction of 20% or more in BALF. The clinical characteristics of the validation cohort are presented in Table 4. Patients were diagnosed in our hospital between 2011 and 2015 for the development cohort and between 2017 and 2018 for the validation cohort. The patients with sarcoidosis tended to be younger than those with other ILDs in both the development and validation cohorts. The diagnosis of each ILD was made according to the relevant criteria.

**Table 3:** Clinical characteristics of the development cohort

	Sarcoidosis	HP	NSIP	COP	DILD	CTD-ILD
Number of patients	18	16	5	5	14	10
Number of lymphocytes analyzed	872	751	253	249	706	637
Age	60.0 ± 3.8	70.9 ± 2.1	73.0 ± 3.0	65.0 ± 6.2	62.4 ± 2.5	61.2 ± 4.0
Gender(M/F)	6/12	12/4	3/2	4/1	11/3	3/7
Smoking (yes/no)	9/9	10/5 (NR: 1)	2/3	3/2	12/2	4/6
Total cell count (*10^4^/ml)	2.12 ± 0.34	3.33 ± 0.76	2.07 ± 0.20	3.56 ± 0.92	2.51 ± 0.46	2.64 ± 0.33
Lymphocyte count (*10^5^/ml)	7.13 ± 1.07	15.4 ± 4.17	8.15 ± 2.00	13.2 ± 4.08	11.9 ± 3.45	13.8 ± 2.94
Fraction of lymphocyte (%)	34.0 ± 2.67	42.0 ± 3.71	38.3 ± 6.09	37.6 ± 10.4	42.1 ± 3.62	50.8 ± 5.82

HP, hypersensitivity pneumonia; NSIP, nonspecific interstitial pneumonia; COP, cryptogenic organizing pneumonia; DILD, drug-induced interstitial lung disease; CTD-ILD, connective tissue disease-associated interstitial lung disease; NR, not reported

**Table 4:** Clinical characteristics of the validation cohort

	Sarcoidosis	Other ILDs
Number of patients	8	45
Number of lymphocytes analyzed	439	2662
Age	56.3 ± 5.0	65.0 ± 2.0
Gender (male/female)	3/5	25/20
Smkoking (yes/no)	4/4	23/21 (NR: 1)
IIPs (COP or AIP)		5
IIPs (uILD)		5
CTD-ILD		12
HP		4
DILD		11
Infection		2
Others		6

ILD, interstitial lung disease; NR, not reported; IIP, idiopathic interstitial pneumonia; COP, cryptogenic organizing pneumonia; AIP, acute interstitial pneumonia; uILD, unclassifiable interstitial lung disease; CTD-ILD, connective tissue disease-associated interstitial lung disease; HP, hypersensitivity pneumonia; DILD, drug-induced interstitial lung disease

### BAL procedure and cell analysis

BAL was performed as previously described [19]. In brief, 50 mL of saline was introduced into the lung and promptly drawn using a bronchoscope. This procedure was repeated three times, and, thus, 150 mL of saline was added. The BAL target region, which was either the right middle lobe or the lingular segment of the left lung, was determined in accordance with the findings of HRCT performed just before BAL. The retrieved saline was centrifuged promptly, and the precipitate was resuspended in the medium, which was followed by cytocentrifugation for 5 min at 600 rpm in a Shandon Cytospin 4 cytocentrifuge (Thermo Fisher Scientific, Waltham, MA. USA). The Diff-Quik staining technique was used to prepare the BALF cell specimens. The cell specimens were reviewed by trained pulmonologists in our department and the differential counts of BALF monocytes, lymphocytes, neutrophils, and eosinophils were determined from at least 500 leukocytes.

### Quantitative assessment of BALF lymphocyte nuclear morphology

High power field (× 400) images for each BALF cell specimen were taken using BIOREVO BZ-9000 (Keyence, Osaka, Japan) and saved as TIFF files with a resolution of 300 dpi. The TIFF files were scanned using Image Pro Plus v6.3 (Media Cybernetics, Inc. Rockville, MD., USA) software. After manually tracing the outlines of the lymphocyte nuclei, the software automatically calculated the area, perimeter, radius ratio, and roundness of each lymphocyte nucleus (Additional file 1: Fig. S5). The radius ratio is the ratio of the maximum radius to the minimum radius. Roundness, which is the measure of how closely the shape of an object approaches that of a circle, is calculated as follows: roundness = (perimeter)^2^/4 × π × area. The roundness for a perfect circle equals 1, while that for a non-circular shape is greater than 1. Therefore, when the roundness value decreases to close to 1.0, it implies “increased roundness”. On the other hand, when the roundness value exceeds 1.0, it implies “decreased roundness”. Approximately 50 lymphocytes from each patient were analyzed. For the validation cohort, clinical information, including the diagnosis of the patients, was blinded during the analysis. As shown in Tables 3 and 4, 3468 lymphocytes of the patients in the development cohort (872 of those with sarcoidosis and 2596 of those with the other ILDs) and 3101 lymphocytes of the patients in the validation cohort (439 of those with sarcoidosis and 2662 of those with other ILDs) were analyzed.

### Statistical analysis

Statistical analyses were performed using SPSS for Windows (version 18.0; SPSS Inc., Chicago, IL, USA). Representative values of continuous variables are presented as mean ± standard error of the mean. Data for individual variables from the two groups were tested using the Mann–Whitney U test, whereas those from three or more groups were tested using the Kruskal–Wallis test followed by multiple comparisons with Bonferroni correction of the significance level. The fraction of lymphocytes with small and/or round nuclei was calculated as the ratio of the count of such lymphocytes to the total lymphocyte count. The comparison of plural AUROC values was performed using EZR software (version 1.53; Saitama Medical Center, Jichi Medical University, Saitama, Japan), which is a graphical user interface for R (The R Foundation for Statistical Computing, Vienna, Austria) [20].

## Supplementary Information

**Additional file 1: Fig. S1**. Frequency distribution of BALF lymphocyte nuclear area. The dark gray bars represent the frequency distribution of the BALF lymphocyte nuclear area in sarcoidosis patients, while the light gray bars represent those in the other ILD patients. The vertical dotted line represents the sarcoidosis-diagnostic threshold of the nuclear area (428.1 μm^2^). Representative lymphocytes with small nuclear areas, those near the threshold value, and those with large nuclear areas are shown in the lower left panel, lower middle panel, and lower right panel, respectively. **Fig. S2**. Frequency distribution of BALF lymphocyte nuclear perimeters. The dark gray bars represent the frequency distribution of the BALF lymphocyte nuclear perimeters in sarcoidosis patients, while the light gray bars represent those in the other ILD patients. The vertical dotted line represents the sarcoidosis-diagnostic threshold of the nuclear perimeter (71.3 μm). Representative lymphocytes with short nuclear perimeters, those near the threshold value, and those with large nuclear perimeters are shown in the lower left panel, lower middle panel, and lower right panel, respectively. **Fig. S3**. Frequency distribution of BALF lymphocyte nuclear radius ratios. The dark gray bars represent the frequency distribution of the BALF lymphocyte nuclear radius ratios in sarcoidosis patients, while the light gray bars represent those in the other ILD patients. The vertical dotted line represents the sarcoidosis diagnostic threshold of the nuclear radius ratio (1.346). Representative lymphocytes with small nuclear radius ratios, those near the threshold value, and those with large nuclear radius ratios are shown in the lower left panel, lower middle panel, and lower right panel, respectively. **Fig. S4**. Frequency distribution of BALF lymphocyte nuclear roundness. The dark gray bars represent the frequency distribution of BALF lymphocyte nuclear roundness in sarcoidosis patients, while the light gray bars represent those in the other ILD patients. The vertical dotted line represents the sarcoidosis diagnostic threshold for nuclear roundness (1.0057). Representative lymphocytes with small nuclear roundness, those near the threshold value, and those with large nuclear roundness are shown in the lower left panel, lower middle panel, and lower right panel, respectively. **Fig. S5**. The four morphological parameters of BALF lymphocyte nuclei. The figures are representing the definition of area, perimeter, radius ratio and roundness of lymphocyte nucleus

## Data Availability

The datasets used and/or analyzed during the current study are available from the corresponding author upon reasonable request.

## Abbreviations

AUROC: Area under the receiver operating characteristic curve
BAL: Bronchoalveolar lavage
BALF: Bronchoalveolar lavage fluid
CHP: Chronic hypersensitivity pneumonia
CTD-ILD: Connective-tissue disease associated interstitial lung disease
HP: Hypersensitivity pneumonia
HRCT: High-resolution computed tomography
ILD: Interstitial lung disease
IPF: Idiopathic pulmonary fibrosis
ROC: Receiver operating characteristic
