# Supplementary material for: Quantitative parameters of lymphocyte nuclear morphology in bronchoalveolar lavage fluid as novel biomarkers for sarcoidosis
Source: Orphanet J Rare Dis. 2021 Jul 3;16:298. doi: 10.1186/s13023-021-01926-x (PMC8254956; doi:10.1186/s13023-021-01926-x)
Supplement: Supplementary file 1 — Additional file 1: Fig. S1. Frequency distribution of BALF lymphocyte nuclear area. The dark gray bars represent the frequency distribution of the BALF lymphocyte nuclear area in sarcoidosis patients, while the light gray bars represent those in the other ILD patients. The vertical dotted line represents the sarcoidosis-diagnostic threshold of the nuclear area (428.1 μm2). Representative lymphocytes with small nuclear areas, those near the threshold value, and those with large nuclear areas are shown in the lower left panel, lower middle panel, and lower right panel, respectively. Fig. S2. Frequency distribution of BALF lymphocyte nuclear perimeters. The dark gray bars represent the frequency distribution of the BALF lymphocyte nuclear perimeters in sarcoidosis patients, while the light gray bars represent those in the other ILD patients. The vertical dotted line represents the sarcoidosis-diagnostic threshold of the nuclear perimeter (71.3 μm). Representative lymphocytes with short nuclear perimeters, those near the threshold value, and those with large nuclear perimeters are shown in the lower left panel, lower middle panel, and lower right panel, respectively. Fig. S3. Frequency distribution of BALF lymphocyte nuclear radius ratios. The dark gray bars represent the frequency distribution of the BALF lymphocyte nuclear radius ratios in sarcoidosis patients, while the light gray bars represent those in the other ILD patients. The vertical dotted line represents the sarcoidosis diagnostic threshold of the nuclear radius ratio (1.346). Representative lymphocytes with small nuclear radius ratios, those near the threshold value, and those with large nuclear radius ratios are shown in the lower left panel, lower middle panel, and lower right panel, respectively. Fig. S4. Frequency distribution of BALF lymphocyte nuclear roundness. The dark gray bars represent the frequency distribution of BALF lymphocyte nuclear roundness in sarcoidosis patients, while the light gray ba [file 13023_2021_1926_MOESM1_ESM.pdf]

Additional File for

"Usefulness of quantitative parameters of lymphocyte nuclear morphology in bronchoalveolar lavage fluid as novel biomarkers for sarcoidosis"

Supplementary Figure 1. Frequency distribution of BALF lymphocyte nuclear area

Supplementary Figure 2. Frequency distribution of BALF lymphocyte nuclear perimeter

Supplementary Figure 3. Frequency distribution of BALF lymphocyte nuclear radius ratio

Supplementary Figure 4. Frequency distribution of BALF lymphocyte nuclear roundness

Supplementary Figure 5. The four morphological parameters of BALF lymphocyte nuclei

Supplementary Figure 1.

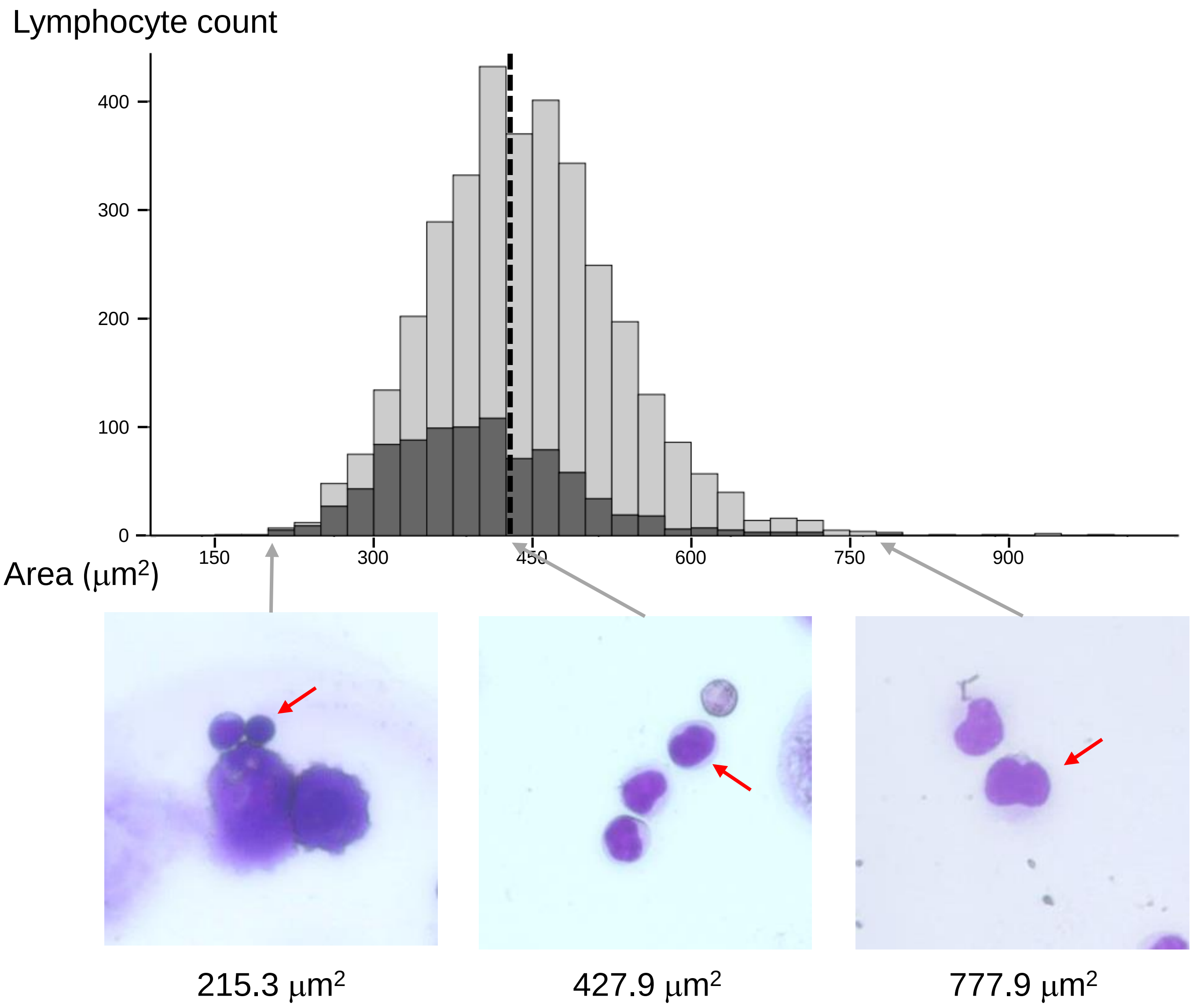

Supplementary Figure 2.

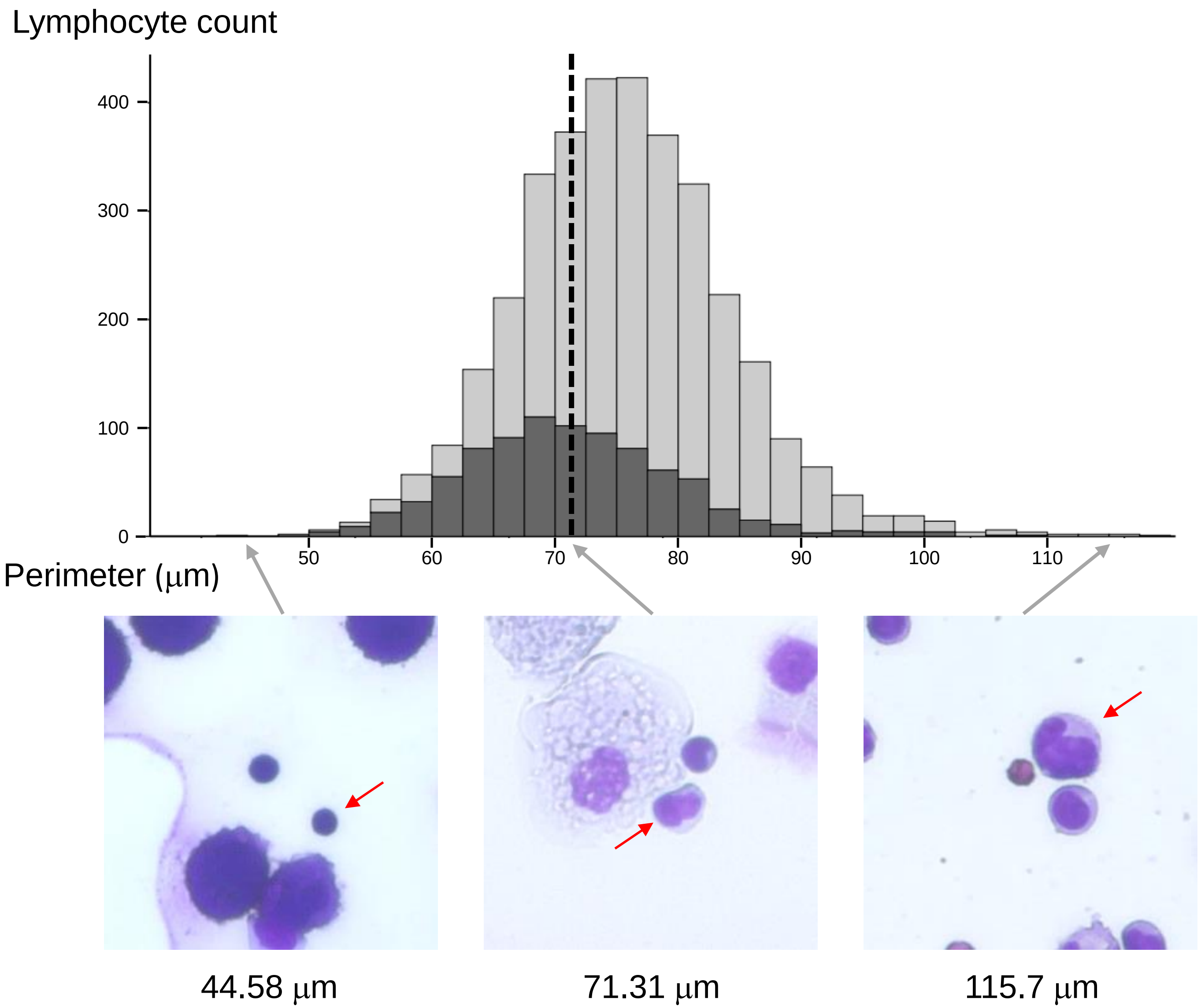

Supplementary Figure 3.

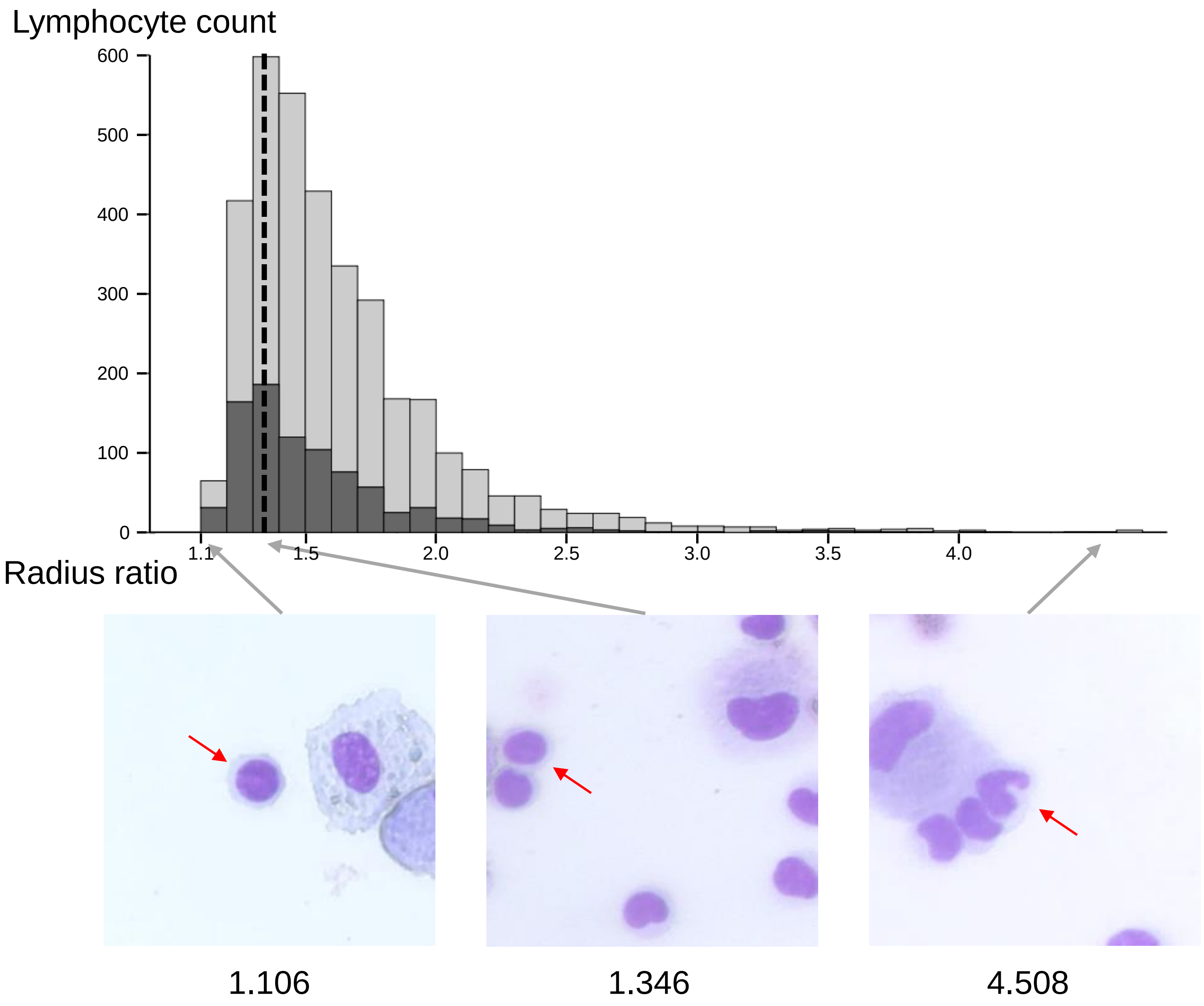

Supplementary Figure 4.

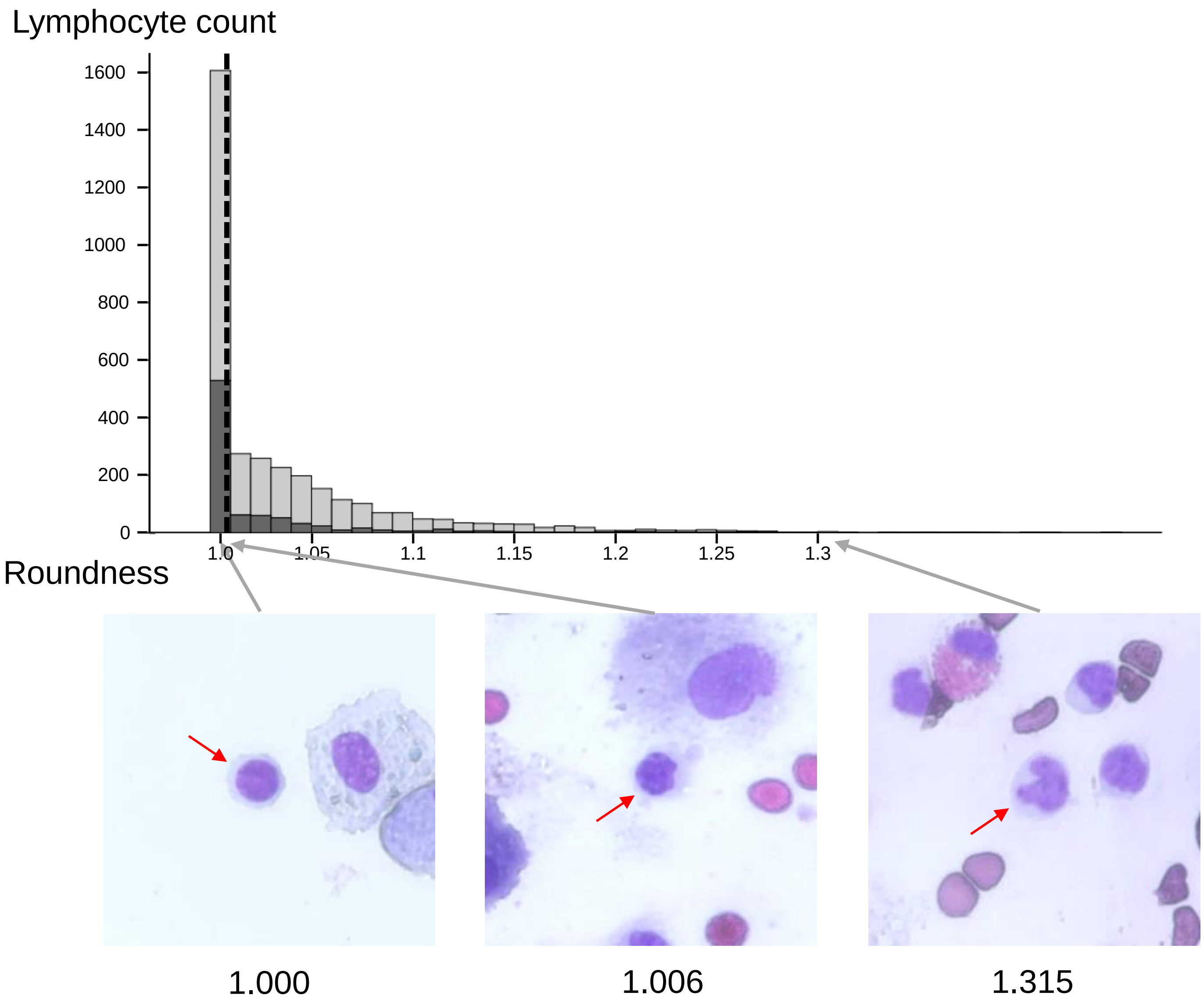

Supplementary Figure 5.

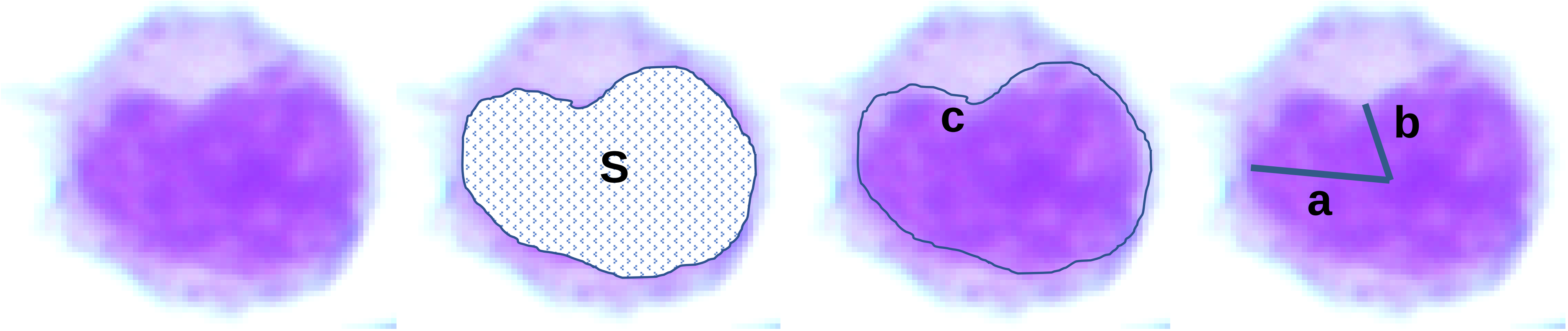

Area = S

Perimeter = c

Radius ratio = a (maximum radius) / b (minimum radius)

Roundness = $c^2 / 4 \times \pi \times S$
